# Deep Learning Models for Multi-Part Morphological Segmentation and Evaluation of Live Unstained Human Sperm

**DOI:** 10.3390/s25103093

**Published:** 2025-05-14

**Authors:** Peiran Lei, Mozafar Saadat, Mahdieh Gol Hassani, Chang Shu

**Affiliations:** Department of Mechanical Engineering, School of Engineering, University of Birmingham, Birmingham B15 2TT, UK; m.saadat@bham.ac.uk (M.S.); mxg238@student.bham.ac.uk (M.G.H.); cxs176@bham.ac.uk (C.S.)

**Keywords:** infertility, sperm morphology, sperm segmentation, deep learning, convolutional neural network

## Abstract

To perform accurate computer vision quality assessments of sperm used within reproductive medicine, a clear separation of each sperm component from the background is critical. This study systematically evaluates and compares the performance of Mask R-CNN, YOLOv8, YOLO11, and U-Net in multi-part sperm segmentation, focusing on the head, acrosome, nucleus, neck, and tail. This study conducts a quantitative analysis using a dataset of live, unstained human sperm, employing multiple metrics, including IoU, Dice, Precision, Recall, and F1 Score. The results indicate that Mask R-CNN outperforms other models in segmenting smaller and more regular structures (head, nucleus, and acrosome). In particular, it achieves a slightly higher IoU than YOLOv8 for the nucleus and surpasses YOLO11 for the acrosome, highlighting its robustness. For the neck, YOLOv8 performs comparably to or slightly better than Mask R-CNN, suggesting that single-stage models can rival two-stage models under certain conditions. For the morphologically complex tail, U-Net achieves the highest IoU, demonstrating the advantage of global perception and multi-scale feature extraction. These findings provide insights into model selection for sperm segmentation tasks, facilitating the optimization of segmentation architectures and advancing applications in assisted reproduction and biological image analysis.

## 1. Introduction

Infertility affects approximately 15% of couples worldwide, and in vitro fertilization (IVF) has become one of the most effective solutions to this issue [1]. In 1992, the field of human reproductive medicine reached a significant milestone with the birth of the first baby conceived via intracytoplasmic sperm injection (ICSI) [2]. In the traditional ICSI process, embryologists must manually select the most motile sperm from a large pool, a process that is highly dependent on visual assessment and professional expertise [3]. However, this manual selection approach is not only time-consuming and labor-intensive but also highly dependent on the operator’s proficiency [4,5]. Any errors during the selection process may result in fertilization failure, leading to the wastage of valuable oocytes [6,7]. Unlike sperm, oocyte retrieval requires invasive and costly procedures [8,9].

**Figure 1:**
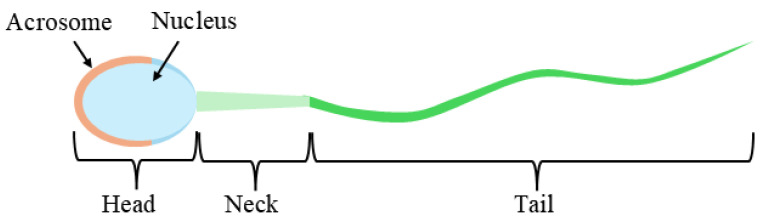
Sperm Structure Diagram.

A mature sperm cell consists of several distinct parts. As shown in Figure 1, the structural diagram of a sperm cell highlights its key components. The head contains the acrosome and the nucleus; the acrosome facilitates penetration of the oocyte for fertilization, while the nucleus carries genetic material [10,11]. The neck provides energy, and the tail enables sperm motility [12]. According to the World Health Organization (WHO) guidelines [13], sperm evaluation includes morphology, motility, and concentration [14]. Any abnormalities in these factors can impair sperm function and ultimately affect fertility.

To address these challenges associated with manual sperm selection, Computer-Aided Sperm Analysis (CASA) systems have emerged as a critical technology in contemporary reproductive medicine [15,16]. These systems provide embryologists with automated tools for sperm selection, making the process more efficient and cost-effective, thereby making IVF more accessible to a broader population [17]. CASA systems can automatically evaluate sperm motility and concentration and have the potential to standardize sperm selection by minimizing the variability associated with manual judgment [18]. However, despite the advancements in CASA systems, even the most advanced versions still require operator intervention for sperm morphology evaluation, introducing potential bias and human error [19,20]. Moreover, precise sperm morphology analysis, especially the accurate segmentation of distinct components such as the head, acrosome, nucleus, neck, and tail, is essential for evaluating sperm quality [21]. The shape and size characteristics of these parts are often key indicators of sperm health and fertility potential [22,23]. Therefore, improving the accuracy and automation of sperm morphology evaluation remains essential [24].

Despite the emergence of CASA systems, precise segmentation of sperm images remains a significant challenge [25]. Factors such as poor image quality, unclear neck, and overlapping sperm heads can complicate the segmentation process [26]. Unlike stained sperm images, segmenting unstained live sperm images presents greater challenges [27]. Staining procedures enhance image contrast, facilitating the distinction of sperm structures [28]. In contrast, unstained images often exhibit low signal-to-noise ratios, indistinct structural boundaries, and minimal color differentiation between components, all of which hinder accurate detection and segmentation [29]. Moreover, staining can alter sperm morphology and structure, potentially compromising their diagnostic value in clinical settings [30]. Therefore, accurate segmentation of unstained sperm is not only technically challenging but also clinically important, as it better reflects real-world applications. In addition, current research often focuses on individual algorithms, stained datasets, or specific regions, which makes it challenging to evaluate the performance of different segmentation techniques under standardized conditions [31,32,33,34,35,36]. Consequently, current studies lack comprehensive segmentation of all sperm components (head, acrosome, nucleus, neck, and tail) within a unified framework, as well as systematic comparisons of various segmentation methods applied to unstained live human sperm datasets.

The aim of this study is to focus on automatic multi-part segmentation (head, acrosome, nucleus, neck, and tail) of unstained live human sperm images within a unified experimental framework. Four deep learning models (Mask R-CNN, U-Net, YOLOv8, and YOLO11) are compared, and their segmentation performance is quantitatively evaluated using metrics such as IoU, Dice, F1 Score, Precision, and Recall. The findings provide new evidence for understanding the applicability and stability of different models across diverse sperm morphologies, laying a foundation for optimizing sperm segmentation models, improving Computer-Aided Sperm Analysis (CASA) systems, and enhancing the accuracy of sperm selection in assisted reproductive technologies such as ICSI.

## 2. Related Work

In the field of sperm analysis and segmentation, numerous studies have introduced a variety of methods aimed at improving accuracy and efficiency. These methods range from traditional image processing techniques to more recent advancements utilizing deep learning. Chang et al. [35] developed a two-stage framework for detecting and segmenting human sperm acrosome and nucleus using k-means clustering and mathematical morphology. Their approach achieved over 98% accuracy in sperm head detection, setting a strong baseline for further work in this area. Similarly, Ghasemian et al. [37] introduced an algorithm focused on detecting morphological abnormalities in sperm images, leveraging size and shape analysis to achieve over 90% accuracy in abnormality detection. These early studies laid the groundwork for segmentation tasks by employing traditional image processing and machine learning techniques.

As deep learning gained prominence, Shaker et al. [32] introduced a fully automated framework for sperm segmentation, using thresholding and edge-based active contour methods. Their method marked a shift towards more robust segmentation, achieving accuracy of 92% for sperm heads, 84% for acrosomes, and 87% for nuclei. Following this, Zhang et al. [38] applied computer vision techniques to animal sperm morphology, using K-means clustering and the Snakes active contour model. These approaches, while innovative, still relied heavily on traditional techniques combined with early deep learning components.

With the rise of convolutional neural networks (CNNs) and transfer learning, more advanced frameworks emerged. Movahed et al. [33] combined CNNs with K-means clustering and SVM classifiers to develop a robust sperm part segmentation framework, significantly improving segmentation accuracy. Prasetyo et al. [39] further compared deep learning models, such as YOLO and Mask R-CNN, for segmenting fish sperm heads and tails, finding that YOLO outperformed Mask R-CNN with a mean average precision of 80.12%. The introduction of specialized neural network architecture led to further advancements in the field. Marín and Chang [31] explored the impact of transfer learning on sperm segmentation, demonstrating that U-Net with transfer learning outperformed Mask R-CNN on the SCIAN-SpermSegGS dataset. Similarly, Lv et al. [40] proposed an improved U-Net model for sperm head segmentation, incorporating hybrid dilated convolutions to achieve high accuracy in complex images.

In terms of dataset contributions, Chen et al. [41] introduced the SVIA dataset, a large-scale resource for sperm analysis tasks, including object detection, segmentation, and tracking. This dataset filled a significant gap in the availability of large, annotated sperm datasets, facilitating further research in the field. Concurrently, Fraczek et al. [42] used Mask R-CNN and SVMs to segment sperm heads and tails and classify head defects, highlighting challenges in flagella segmentation.

Recently, Suleman et al. [43] reviewed various deep learning techniques for sperm fertility prediction, identifying CNNs as particularly effective in sperm morphology analysis. Lewandowska et al. [26] took a more ensemble-based approach, combining multiple segmentation algorithms to handle noisy and blurry sperm images. Their method showed promise in addressing challenges posed by low-quality inputs.

In 2024, two important contributions further advanced the field. Chen et al. [34] introduced the Cell Parsing Net (CP-Net), which integrates instance-aware and part-aware segmentation into a unified framework, achieving superior performance in segmenting tiny subcellular structures like sperm acrosomes and midpieces. Their CP-Net model set a new benchmark in sperm segmentation, aided by the introduction of a new sperm parsing dataset. Meanwhile, Yang et al. [44]. developed a multidimensional morphological analysis framework that tracks live sperm using improved FairMOT and segments them with BlendMask and SegNet. This method achieves over 90% accuracy in unstained sperm morphology detection and is particularly valuable for simultaneously analyzing sperm motility and morphology in real time, providing crucial support for intracytoplasmic sperm injection (ICSI) procedures.

In parallel with these domain-specific developments, recent advances in general medical image segmentation have introduced more sophisticated architectures such as Attention U-Net, ResUNet, TransUNet, and SwinUNet [45,46,47,48]. These models incorporate mechanisms like attention, residual connections, and transformers to improve performance in various biomedical segmentation tasks.

In conclusion, these previous works can be broadly categorized into traditional image processing approaches, early CNN-based segmentation methods, and recent advancements using transfer learning, ensemble techniques, and emerging architectures such as transformers and attention-based networks. This progression highlights the shift from traditional machine learning to more complex, deep learning models that continue to push the boundaries of accuracy and applicability in sperm segmentation and morphological analysis. The development of different datasets and more sophisticated architectures has opened new possibilities for both clinical applications and research advancements in the field.

## 3. Methodology

### 3.1. Dataset

In this study, a clinically labeled live, unstained human sperm dataset was used [49,50]. To enable the segmentation model to analyze normal sperm consistently identified by three sperm morphology experts with over 10 years of experience, 93 images labeled as “Normal Fully Agree Sperms” from the dataset were used. Each part of the sperm in every image (acrosome, nucleus, head, midpiece, and tail) was accurately annotated. These annotations were then paired with their corresponding images and divided into training and validation sets, with 20% allocated to the training set and 80% to the validation set. This split ensures a reliable evaluation of the model’s performance.

### 3.2. Pre-Processing

To enhance the robustness and generalization capability of the model, a series of data augmentation techniques were employed in this study. These techniques included horizontal and vertical flipping with a 50% probability to introduce diversity in object orientation, thereby enabling the model to remain invariant to such variations. Additionally, brightness and contrast adjustments were applied, with brightness changes limited to 30% and contrast changes limited to 20%. These adjustments were applied with a certain probability to simulate varying lighting conditions.

A series of transformations was applied to enhance the diversity of the training dataset, including simulating image compression to mitigate quality loss, random gamma correction for introducing nonlinear variations in brightness and contrast, blur effects to emulate out-of-focus images, and histogram equalization for contrast enhancement. These diverse augmentation methods help the model adapt to variations in image quality, lighting conditions, and object orientation, thereby improving its performance and robustness in practical applications. Additionally, the images were normalized using specific mean values (0.485, 0.456, 0.406) and standard deviations (0.229, 0.224, 0.225). Normalization centralizes pixel values and scales them appropriately, accelerating model convergence and enhancing training stability.

Following these pre-processing steps, the augmented dataset was found to meet the fundamental data requirements for deep learning applications. Consistent and promising results across multiple runs further demonstrate the robustness and generalization capability of the models trained on this dataset.

### 3.3. Overview of the Four Methods

**Figure 2:**
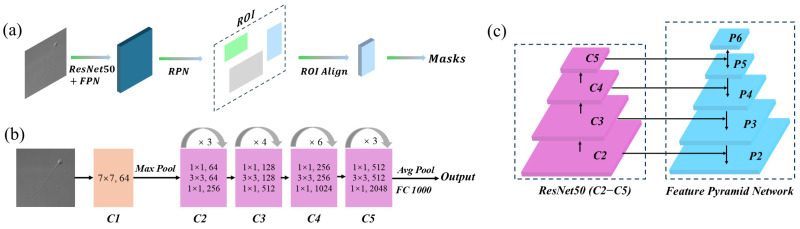
Mask R-CNN Architecture. (**a**) Mask R-CNN Main Architecture; (**b**) ResNet50 Backbone Structure; (**c**) Feature Pyramid Network.

This study utilizes four methods to perform segmentation on different parts of the sperm. The first method is Mask R-CNN, which generates corresponding segmentation masks for each detected part of the sperm [51]. Figure 2 illustrates the overall structure of our Mask R-CNN implementation, where Figure 2a presents the main structure, Figure 2b details the ResNet50 backbone, and Figure 2c illustrates the Feature Pyramid Network (FPN) used for multi-scale feature extraction. The workflow begins by inputting sperm images and their corresponding annotations into a convolutional neural network, specifically utilizing the maskrcnn_resnet50_fpn model for training [52]. During the image processing stage, Mask R-CNN employs ResNet-50 as the feature extractor. ResNet-50, a critical component of the model, consists of 50 convolutional layers and is primarily composed of an initial convolutional layer, pooling layers, residual blocks, and the overall network architecture.

**Figure 3:**
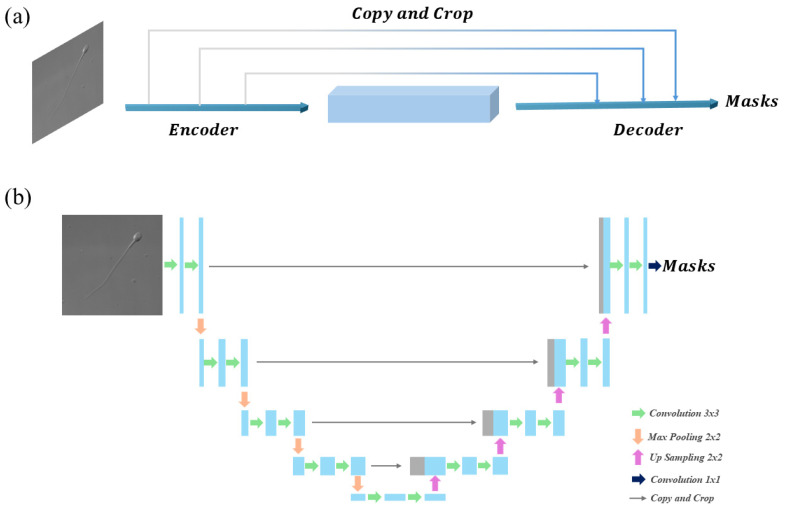
U-Net Architecture. (**a**) U-Net Main Architecture; (**b**) U-Net Detailed Operations.

The second approach involves using U-Net, a fully convolutional network specifically designed for medical image segmentation [53]. Figure 3 illustrates the U-Net architecture, where Figure 3a represents the main encoder–decoder structure, and Figure 3b provides a detailed breakdown of convolutional, pooling, and upsampling operations used for segmentation. The U-Net architecture consists of three main components: the encoder path, the decoder path, and the output layer [54]. When sperm images are input into the network, they are progressively processed by the encoder to generate feature maps. These feature maps are then concatenated in the decoder, culminating in the output of segmentation masks for the targeted regions. Named for its characteristic U-shaped structure, U-Net features an encoder on the left, a decoder on the right, and skip connections that link intermediate layers.

**Figure 4:**
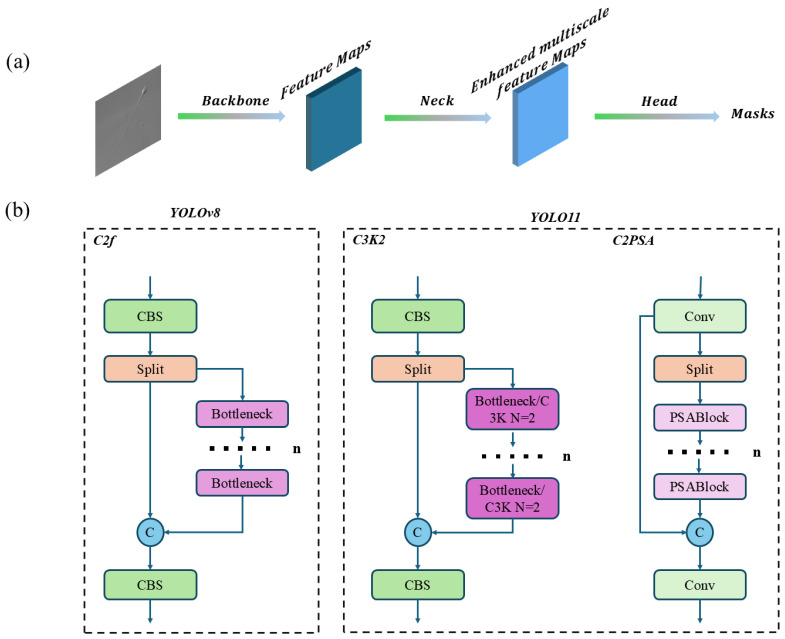
YOLO(v8/11) Architecture. (**a**) YOLO Main Architecture; (**b**) YOLOv8 and YOLO11 Architectural Comparison.

YOLOv8 and YOLO11 are recent versions of the YOLO series. In addition to object detection, these models also provide segmentation capabilities. As this work focuses solely on segmentation, the terms YOLOv8 and YOLO11 refer specifically to their segmentation variants, YOLOv8-seg and YOLO11-seg. These models are extensively utilized in various studies for their exceptional speed and accuracy [55,56]. Figure 4 illustrates the YOLO architecture. Figure 4a presents the general main structure of YOLO, consisting of the Backbone, Neck, and Head components. Figure 4b highlights the architectural differences between YOLOv8 and YOLO11. In the Backbone, YOLOv8 utilizes CSPNet, while YOLO11 modifies this by substituting C2f with C3K2 and incorporating an additional C2PSA layer at the end [57]. Regarding the Head, YOLOv8 employs an anchor-free mechanism, whereas YOLO11 introduces a modification by replacing CV3 in the detection head with parallel DWConv processing.

### 3.4. Evaluation Metrics

Different performance metrics are used to evaluate segmentation performance. Some serve as primary metrics, some as secondary metrics, and others as auxiliary metrics. In segmentation tasks, the most commonly used evaluation metrics are IoU, also known as the Jaccard Index, and the Dice coefficient shown as the Equations (1) and (2) [58]. IoU measures the degree of overlap between the predicted region and the ground truth region. The Dice coefficient illustrates the relationship between the non-overlapping and overlapping parts.

(1) $$IoU=\frac{TP}{TP+FP+FN}$$

(2) $$Dice=\frac{2\cdot TP}{2\cdot TP+FP+FN}$$

where TP (True positive) represents the number of pixels correctly segmented by the prediction, FP (False Positive) represents the number of pixels mistakenly segmented from non-ground truth regions, and FN (False Negative) represents the number of ground truth pixels that were missed and left unsegmented.

Furthermore, Precision and Recall can be utilized as pixel-level evaluation metrics to assess the performance of segmentation results [58,59]. These metrics are calculated based on the values derived from the Confusion Matrix and are shown as Equations (3) and (4) [33].

(3) $$Precision=\frac{TP}{TP+FP}$$

(4) $$Recall=\frac{TP}{TP+FN}$$

where TP (True positive) represents the number of pixels correctly segmented by the prediction, FP (False Positive) represents the number of pixels mistakenly segmented from non-ground truth regions, and FN (False Negative) represents the number of ground truth pixels that were missed and left unsegmented.

The harmonic mean of Precision and Recall, known as the F1 Score, provides a comprehensive and intuitive assessment of these metrics [58].

## 4. Results

In this section, four different methods were used to segment unstained sperm, and their performance was compared. These methods include Mask R-CNN, U-Net, YOLOv8, and YOLO11. Among them, YOLOv8, YOLO11, and U-Net are one-stage image segmentation models, while Mask R-CNN is a two-stage image segmentation model. In Results and Discussions, all the reported metrics (IoU, Dice, Precision, Recall, F1) represent mean values. Experimental results show that the Dice coefficient and F1 score yield identical values in binary segmentation tasks. Although their theoretical interpretations differ, both are meaningful for performance evaluation. To avoid redundancy, only the Dice coefficient is presented in the tables.

### 4.1. Head

**Figure 5:**
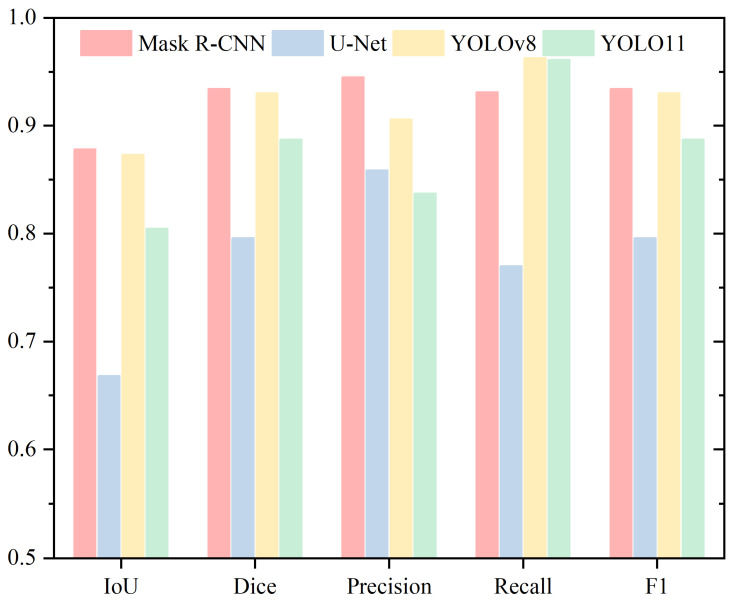
Performance Evaluation of the Head Segmentation Using Mean IoU, Mean Dice, Mean Precision, Mean Recall, and Mean F1 Metrics.

To provide a comprehensive analysis of sperm head segmentation, this study evaluates four methods using five metrics. Table 1 and Figure 5 present the Mean IoU, Mean Dice, Mean Precision, Mean Recall, and Mean F1 metrics for the Head region. Mask R-CNN demonstrates the best performance in both IoU and Dice coefficient, followed by YOLOv8, while U-Net shows the weakest performance. Specifically, in terms of IoU, Mask R-CNN achieves a slight improvement of 0.52% over YOLOv8, 7.34% over YOLO11, and 21.01% over U-Net. For Dice coefficient, Mask R-CNN surpasses YOLOv8 by 0.37%, YOLO11 by 4.67%, and U-Net by 13.83%.

The IoU and Dice metrics reveal that Mask R-CNN and YOLOv8 exhibit very similar performance, with differences of just 0.52% and 0.37%, respectively. In contrast, U-Net performs significantly worse than Mask R-CNN on both metrics. For Precision, Mask R-CNN demonstrates the highest performance, achieving 94.51%, while YOLO11 records the lowest at 83.74%. YOLOv8 and U-Net rank in the middle with Precision values of 90.60% and 85.89%, respectively. Regarding Recall, the order from highest to lowest is YOLOv8 (96.28%), YOLO11 (96.13%), Mask R-CNN (93.12%), and U-Net (77.01%). For the F1 score, the ranking is Mask R-CNN (93.42%), YOLOv8 (93.05%), YOLO11 (88.75%), and U-Net (79.59%).

The rankings for Precision, Recall, and F1 Score differ across these evaluation metrics. For Precision, Mask R-CNN achieves the highest performance, exceeding YOLO11, the lowest performer, by 10.77%. In Recall, YOLOv8 ranks highest, outperforming U-Net by 19.27%. However, the performance gap between YOLOv8 and the second-highest YOLO11 is relatively small, at just 0.15%. Regarding the F1 Score, Mask R-CNN and YOLOv8 exhibit similar performance, differing by only 0.37%.

### 4.2. Acrosome

**Figure 6:**
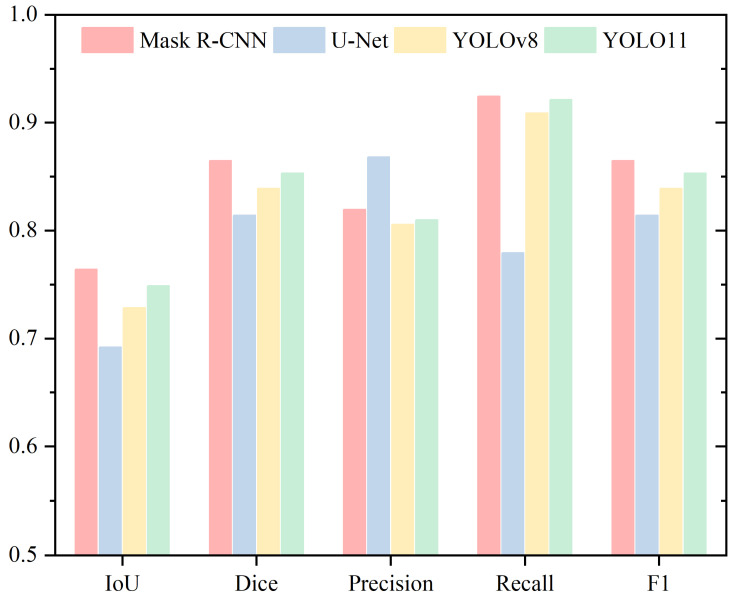
Performance Evaluation of Acrosome Segmentation Using Mean IoU, Mean Dice, Mean Precision, Mean Recall, and Mean F1 Metrics.

For acrosome segmentation, Table 2 and Figure 6 present the Mean IoU, Mean Dice, Mean Precision, Mean Recall, and Mean F1 metrics. The IoU and Dice metrics show a consistent trend, with Mask R-CNN achieving the highest performance, followed by YOLO11, YOLOv8, and U-Net. The IoU values, in descending order, are 76.41%, 74.87%, 72.84%, and 69.20%. Similarly, the Dice values, in descending order, are 86.48%, 85.31%, 83.90%, and 81.42%.

Mask R-CNN leads in the IoU metric, with a minimal difference of 1.54% compared to the second-best YOLO11, suggesting their performance is closely matched. In contrast, it outperforms U-Net, the lowest-performing model, by 7.21%, highlighting that Mask R-CNN delivers the best performance for acrosome segmentation.

For the Dice metric, the performance differences among the models are relatively small. Mask R-CNN and YOLO11 show a difference of 1.17%, while YOLO11 and YOLOv8 differ by 1.41%, whereas the difference between YOLOv8 and U-Net is 2.48%. Overall, the gap between the top-performing Mask R-CNN and the lowest-performing U-Net is 5.06%, with YOLO11 and YOLOv8 positioned within this range. For Precision, the models rank from highest to lowest as U-Net, Mask R-CNN, YOLO11, and YOLOv8. In both Recall and F1 Score, the ranking order is Mask R-CNN, YOLO11, YOLOv8, and U-Net.

### 4.3. Nucleus

**Figure 7:**
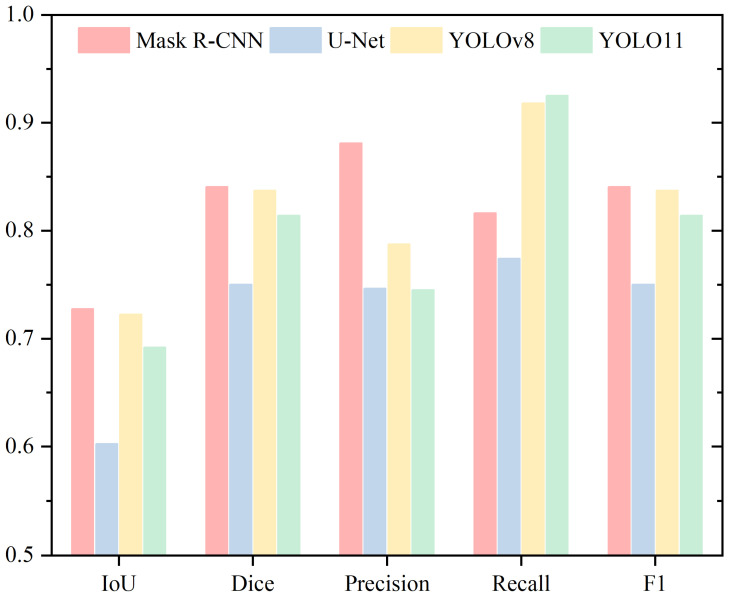
Performance Evaluation of Nucleus Segmentation Using Mean IoU, Mean Dice, Mean Precision, Mean Recall, and Mean F1 Metrics.

For nucleus segmentation, Table 3 and Figure 7 present the Mean IoU, Mean Dice, Mean Precision, Mean Recall, and Mean F1 metrics. Mask R-CNN achieves the highest scores for both IoU and Dice, with slight differences of 0.51% and 0.35% compared to YOLOv8, respectively. In contrast, U-Net records the lowest scores in these metrics, exhibiting a substantial gap of 12.49% and 9.06% compared to Mask R-CNN. Mask R-CNN attains the highest scores in Precision and F1 Score, whereas YOLO11 achieves the best performance in Recall.

### 4.4. Neck

**Figure 8:**
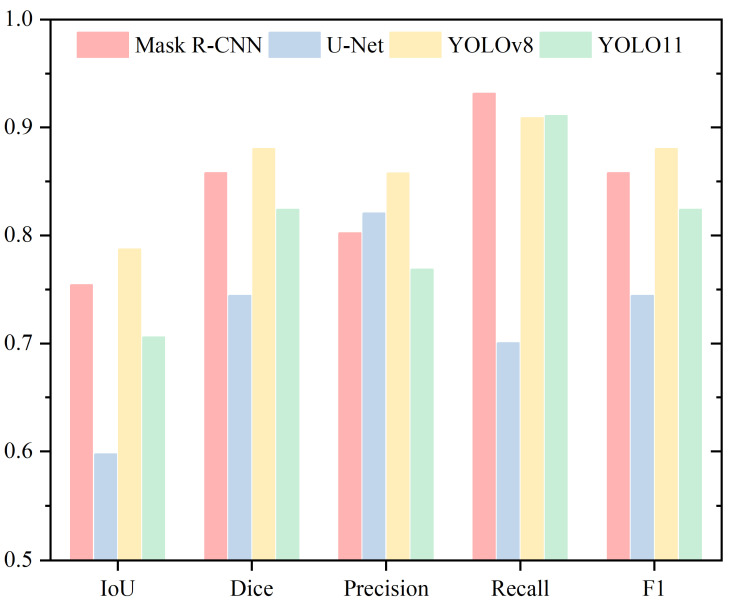
Performance Evaluation of Neck Segmentation Using Mean IoU, Mean Dice, Mean Precision, Mean Recall, and Mean F1 Metrics.

In the analysis of the neck, Table 4 and Figure 8 present the Mean IoU, Mean Dice, Mean Precision, Mean Recall, and Mean F1 metrics. Both IoU and Dice metrics follow the same trend, ranking the models from highest to lowest as YOLOv8, Mask R-CNN, YOLO11, and U-Net. YOLOv8 and Mask R-CNN show similar performance, with differences of 3.30% and 2.24%, respectively. U-Net records the lowest values, showing a substantial gap, with IoU and Dice differences of 18.96% and 13.6% compared to the highest-performing YOLOv8.

For Precision, the models rank from highest to lowest as YOLOv8, U-Net, Mask R-CNN, and YOLO11. Regarding Recall, the rankings are Mask R-CNN, YOLO11, YOLOv8, and U-Net. YOLOv8 and YOLO11 display very similar Recall values, differing by only 0.19%. In contrast, U-Net scores significantly lower than the other three models, with differences of 23.09%, 21.01%, and 20.82%, respectively. For the F1 Score, the ranking is YOLOv8, Mask R-CNN, YOLO11, and U-Net, with a similar trend observed for IoU and Dice.

### 4.5. Tail

**Figure 9:**
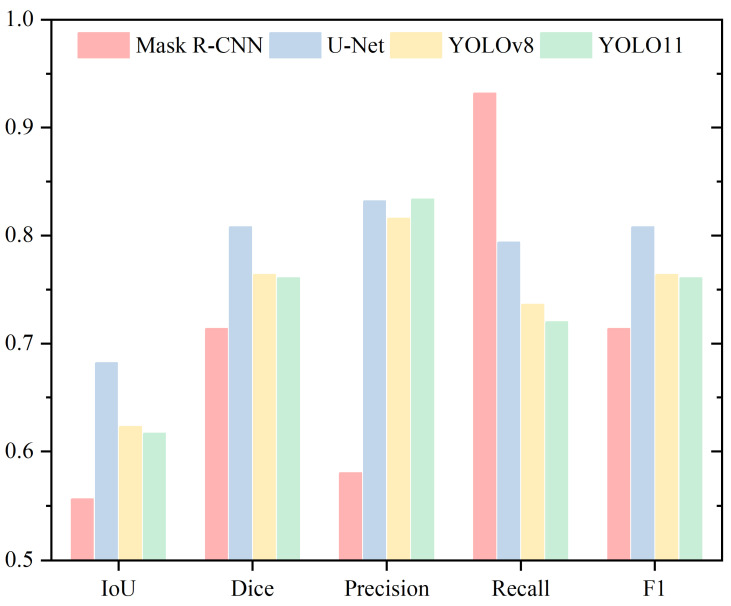
Performance Evaluation of Tail Segmentation Using Mean IoU, Mean Dice, Mean Precision, Mean Recall, and Mean F1 Metrics.

For tail segmentation, Table 5 and Figure 9 present the Mean IoU, Mean Dice, Mean Precision, Mean Recall, and Mean F1 metrics. In the IoU and Dice metrics, a notable observation emerges in the tail region. Unlike the previous four parts (head, acrosome, nucleus, and neck), U-Net, which consistently ranked the lowest, now achieves the highest score, while Mask R-CNN drops to the lowest. Meanwhile, YOLOv8 and YOLO11 show very similar results, with a slight difference of 0.61% and 0.32%, respectively.

In Precision, YOLO11 achieves the highest score, closely followed by U-Net, with a minimal difference of 0.18%. Mask R-CNN lags significantly behind the other three models, with gaps of 25.35%, 25.17%, and 23.57%, respectively. For Recall, Mask R-CNN stands out as the top performer, markedly surpassing the other three models by margins of 21.18%, 19.58%, and 13.81%, respectively. YOLOv8 and YOLO11 show similar performance in Recall, differing by only 1.6%. Regarding the F1 Score, U-Net records the highest value at 80.79%, with YOLOv8 slightly exceeding YOLO11 by 0.32%.

## 5. Discussion

**Figure 10:**
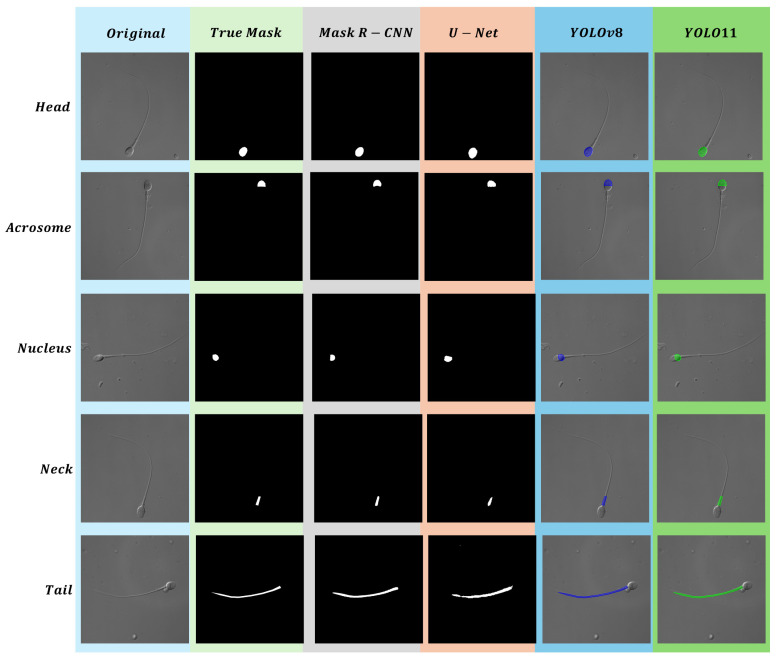
Segmentation Results for Different Parts (Head, Acrosome, Nucleus, Neck, Tail) Across Original, True Masks, Mask R-CNN, U-Net, YOLOv8, and YOLO11.

In this work, four segmentation methods for five sperm parts (head, acrosome, nucleus, neck, and tail) are compared. The performance metrics of different segmentation methods for each part are analyzed and compared. All models achieved inference times below 0.02 s per image on the same hardware, indicating potential for real-time applications. As speed differences were minimal, the analysis focused on segmentation performance. In Figure 10, some segmentation results are displayed, showing the head, acrosome, nucleus, neck, and tail from top to bottom. To discuss the results, this paper categorizes evaluation metrics into three types based on their calculation methods and analyzes the results accordingly. The IoU and Dice coefficient are the most important core metrics, as most studies utilize these two metrics or similar metrics for analysis [60,61]. The secondary metric is the F1 Score, which evaluates the balance between Precision and Recall. Since both Precision and Recall are crucial in segmentation tasks, the F1 Score serves as an optimal comprehensive measure. Finally, the auxiliary metrics are Precision and Recall. Precision indicates the number of pixels incorrectly segmented as the target class, while Recall reflects the number of pixels belonging to the target class that were not detected. These two metrics are able to provide a better demonstration of the segmentation accuracy and can also be combined to calculate the F1 Score. This enables a structured evaluation across anatomically distinct regions and highlights how segmentation performance varies with morphological complexity. In addition, the region-specific comparison provides a practical analytical framework for assessing segmentation models in relation to biological structure, which may be extended to other biomedical imaging tasks.

An analysis of the core metrics, IoU and Dice, shows that Mask R-CNN achieves the highest performance for the head, acrosome, and nucleus parts, while U-Net performs the worst. However, for the tail, the results are completely reversed, with U-Net achieving the highest performance and Mask R-CNN the lowest. The reason for this discrepancy is that parts like the head, acrosome, nucleus, and neck are smaller and have more regular shapes compared to the tail. Methods like Mask R-CNN perform better in segmenting smaller and more regular shapes. The reason for this outcome lies in the use of ROI Align in Mask R-CNN, which differs from the quantization operation in ROI Pooling [62]. ROI Align ensures that detailed features are preserved without being lost due to quantization, making it particularly effective for smaller and more regularly shaped parts. Additionally, ROI Align focuses on the candidate boxes generated by the Region Proposal Network, which means it can concentrate more on the target areas. These reasons lead to better segmentation of boundaries in regular shapes, where the edges are clearer and more well-defined. In contrast, U-Net operates differently by using a full-image feature extraction approach, which makes it less effective than Mask R-CNN in segmenting smaller shapes [63]. Additionally, U-Net’s layer-by-layer down-sampling method can result in blurred boundaries for regular shapes. Additionally, due to its global context characteristics, U-Net is more likely than Mask R-CNN to incorporate background, which can lead to background areas being mistakenly segmented as part of the target, resulting in segmentation errors.

Although Mask R-CNN performs well with small and regular shapes, it performs the worst among the four methods for the tail, which has a slender and irregular shape. This is mainly because the regions in ROI Align struggle to fully cover elongated targets. Additionally, the complex shape of the tail makes it more challenging to extract local features effectively. For these reasons, Mask R-CNN tends to exhibit incomplete detection when segmenting the tail. On the other hand, U-Net’s combination of global context awareness, skip connections, and multi-scale features makes it better suited for segmenting diverse and elongated targets [63,64].

An analysis combining the core metrics IoU and Dice with the secondary metric F1 Score reveals that, for the head and nucleus parts, all metrics indicate that Mask R-CNN slightly outperforms YOLOv8, with all metric differences being less than 0.6%. This demonstrates that YOLOv8 achieves good segmentation performance in these two parts, approaching the performance of the two-stage model Mask R-CNN [65]. The strong performance of YOLOv8 in these two regions highlights its efficiency in maintaining competitive accuracy while benefiting from a streamlined, single-stage, anchor-free architecture, which simplifies model design and facilitates easier deployment in segmentation tasks [66]. For the acrosome part, Mask R-CNN performs the best, while YOLO11 is close, with all metric differences being less than 1.6%. The near parity in performance suggests that the architectural enhancements in YOLO11, such as the adoption of C3K2 modules and C2PSA attention mechanisms, may provide improved feature extraction capabilities compared to previous versions, particularly in more complex structures. In the neck part, all metrics show that YOLOv8 performs the best. This further supports the robustness of its lightweight yet effective design, which balances segmentation accuracy with architectural simplicity. This may be attributed to the shared single-stage design of the model, which enables efficient local feature capture—an essential factor for accurately identifying small and well-localized structures such as the neck [67]. For the tail part, U-Net consistently performs the best, while YOLOv8 and YOLO11 perform very similarly. The consistency in their results indicates that both YOLO variants are capable of handling elongated and less structured regions effectively. From the above analysis, YOLOv8 and 11 demonstrate consistently stable performance across different parts, achieving results that are either close to the best-performing method or represent the best method themselves.

While this study provides a comprehensive comparison of four segmentation methods for sperm part segmentation, ensuring a fair evaluation across different studies remains a challenge. In the field of computer vision, fair comparisons require consistency in datasets and evaluation metrics [68,69,70]. However, since different studies use different datasets, including public sperm datasets such as SCIAN-SpermSegGS, HuSHem, MHSMA and VISEM, which vary in image quality and annotation methods, direct comparisons could lead to unreliable findings [31,71,72,73,74]. To improve comparability under such circumstances, future work should focus on expanding datasets with more diverse samples, applying cross-validation strategies, and conducting evaluations within the same dataset to enhance the robustness, reliability, and fairness of performance assessments. Further efforts may also involve incorporating additional models and developing novel network architectures, with the aim of improving overall experimental outcomes.

## 6. Conclusions

Accurate segmentation is a critical prerequisite for successful computer vision-based detection of sperm structures used in reproductive medicine, such as intracytoplasmic sperm injection. This study systematically evaluated and compared the performance of Mask R-CNN, YOLOv8, YOLO11, and U-Net for multi-part segmentation of human sperm, focusing on accurately segmenting the head, acrosome, nucleus, neck, and tail. The findings provide a quantitative basis for selecting models and optimizing segmentation performance. Mask R-CNN demonstrates superior performance in segmenting smaller and regular structures such as the head and acrosome. U-Net and YOLO-based models exhibit strong potential for handling complex and irregular morphologies. YOLOv8 and YOLO11 demonstrate consistently stable segmentation performance across different sperm parts. The results contribute to the advancements in computer-aided sperm analysis (CASA) systems by comparing and analyzing sperm morphological structures.

## Figures and Tables

**Table 1:** Performance Evaluation of the Head Segmentation Using Mean IoU, Mean Dice, Mean Precision, and Mean Recall.

Head				
	IoU	Dice	Precision	Recall
Mask R-CNN	0.8783	0.9342	0.9451	0.9312
U-Net	0.6682	0.7959	0.8589	0.7701
YOLOv8	0.8731	0.9305	0.9060	0.9628
YOLO11	0.8049	0.8875	0.8374	0.9613

**Table 2:** Performance Evaluation of Acrosome Segmentation Using Mean IoU, Mean Dice, Mean Precision and Mean Recall.

Acrosome				
	IoU	Dice	Precision	Recall
Mask R-CNN	0.7641	0.8648	0.8194	0.9243
U-Net	0.6920	0.8142	0.8681	0.7793
YOLOv8	0.7284	0.8390	0.8056	0.9088
YOLO11	0.7487	0.8531	0.8097	0.9213

**Table 3:** Performance Evaluation of Nucleus Segmentation Using Mean IoU, Mean Dice, Mean Precision, and Mean Recall.

Nucleus				
	IoU	Dice	Precision	Recall
Mask R-CNN	0.7275	0.8408	0.8811	0.8163
U-Net	0.6026	0.7502	0.7466	0.7742
YOLOv8	0.7224	0.8373	0.7875	0.9182
YOLO11	0.6921	0.8142	0.7453	0.9251

**Table 4:** Performance Evaluation of Neck Segmentation Using Mean IoU, Mean Dice, Mean Precision, and Mean Recall.

Neck				
	IoU	Dice	Precision	Recall
Mask R-CNN	0.7542	0.8579	0.8023	0.9315
U-Net	0.5976	0.7443	0.8207	0.7006
YOLOv8	0.7872	0.8803	0.8577	0.9088
YOLO11	0.7058	0.8241	0.7685	0.9107

**Table 5:** Performance Evaluation of Tail Segmentation Using Mean IoU, Mean Dice, Mean Precision, and Mean Recall.

Tail				
	IoU	Dice	Precision	Recall
Mask R-CNN	0.5563	0.7138	0.5802	0.9318
U-Net	0.6821	0.8079	0.8319	0.7937
YOLOv8	0.6229	0.7638	0.8159	0.7360
YOLO11	0.6168	0.7606	0.8337	0.7200

## Data Availability

Publicly available dataset was utilized in this study. Dataset link: https://doi.org/10.26180/25621500.
